# Pharmacological effects of methylone and MDMA in humans

**DOI:** 10.3389/fphar.2023.1122861

**Published:** 2023-02-17

**Authors:** Lourdes Poyatos, Clara Pérez-Mañá, Olga Hladun, Melani Núñez-Montero, Georgina de la Rosa, Soraya Martín, Ana Maria Barriocanal, Lydia Carabias, Benjamin Kelmendi, Omayema Taoussi, Francesco Paolo Busardò, Francina Fonseca, Marta Torrens, Simona Pichini, Magí Farré, Esther Papaseit

**Affiliations:** ^1^ Department of Clinical Pharmacology, Hospital Universitari Germans Trias i Pujol and Institut de Recerca Germans Trias i Pujol (HUGTiP-IGTP), Badalona, Spain; ^2^ Department of Pharmacology, Therapeutics and Toxicology, Universitat Autònoma de Barcelona (UAB), Cerdanyola del Vallés, Spain; ^3^ Department of Pharmacy, Hospital Universitari Germans Trias i Pujol (HUGTiP), Badalona, Spain; ^4^ Department of Psychiatry, Yale University School of Medicine, New Haven, CT, United States; ^5^ Department of Excellence-Biomedical Sciences and Public Health, Università Politecnica delle Marche, Ancona, Italy; ^6^ Department of Medicine and Life Sciences (MELIS), Universitat Pompeu Fabra, Barcelona, Spain; ^7^ Addiction Program, Institut de Neuropsiquiatria i Adiccions (INAD), Barcelona, Spain; ^8^ Department of Psychiatry and Forensic Medicine, Universitat Autònoma de Barcelona (UAB), Cerdanyola del Vallés, Spain; ^9^ National Centre on Addiction and Doping, Istituto Superiore di Sanità, Rome, Italy

**Keywords:** methylone, 3,4-methylenedioxymethcathinone, MDMA, 3,4-methylenedioxymethamphetamine, new psychoactive substances, synthetic cathinones, bath salts, psychostimulants

## Abstract

Methylone is one of the most common synthetic cathinones popularized as a substitute for 3,4-methylenedioxymethamphetamine (MDMA, midomafetamine) owing to its similar effects among users. Both psychostimulants exhibit similar chemistry (i.e., methylone is a β-keto analog of MDMA) and mechanisms of action. Currently, the pharmacology of methylone remains scarcely explored in humans. Herein, we aimed to evaluate the acute pharmacological effects of methylone and its abuse potential in humans when compared with that of MDMA following oral administration under controlled conditions. Seventeen participants of both sexes (14 males, 3 females) with a previous history of psychostimulant use completed a randomized, double-blind, placebo-controlled, crossover clinical trial. Participants received a single oral dose of 200 mg of methylone, 100 mg of MDMA, and a placebo. The variables included physiological effects (blood pressure, heart rate, oral temperature, pupil diameter), subjective effects using visual analog scales (VAS), the short form of the Addiction Research Center Inventory (ARCI), the Evaluation of Subjective Effects of Substances with Abuse Potential questionnaire (VESSPA-SSE), and the Sensitivity to Drug Reinforcement Questionnaire (SDRQ), and psychomotor performance (Maddox wing, psychomotor vigilance task). We observed that methylone could significantly increase blood pressure and heart rate and induce pleasurable effects, such as stimulation, euphoria, wellbeing, enhanced empathy, and altered perception. Methylone exhibited an effect profile similar to MDMA, with a faster overall onset and earlier disappearance of subjective effects. These results suggest that abuse potential of methylone is comparable to that of MDMA in humans.

**Clinical Trial Registration:** https://clinicaltrials.gov/ct2/show/NCT05488171; Identifier: NCT05488171.

## 1 Introduction

Methylone (3,4-methylenedioxy-*N*-methylcathinone), also known as MDMC, βk-MDMA, or M1, is a synthetic cathinone that emerged in 2004 as a new psychoactive substance (NPS), although first synthesized in 1996 and planned for therapeutic use as an antidepressant and potential treatment for the Parkinson’s disease (Bossong et al., 2005). NPSs appear in the market as legal alternatives to common drugs of abuse, benefiting from their status as non-controlled substances by the International Drug Control Conventions. Users can acquire these substances *via* the Internet, advertised as bath salts, plant fertilizers, or incense. Methylone has been popularized as an alternative to MDMA (3,4-methylenedioxymethamphetamine, midomafetamine), which is chemically similar, given that methylone is considered its β-keto analog, differing only by an oxygen atom added at the benzylic position (Bossong et al., 2005).

Similar to MDMA, methylone is a substrate for the high-affinity monoamine transporters–i.e., the dopamine transporter (DAT), norepinephrine transporter (NET), and serotonin (5-HT) transporter (SERT) (Simmler et al., 2011; Sogawa et al., 2011; Baumann et al., 2012; Eshleman et al., 2013; Simmler et al., 2013; Elmore et al., 2017; Luethi et al., 2019). *In vitro* studies from rat brain tissue demonstrate that methylone has a similar mechanism of action as MDMA but is less potent at all three transporters (Baumann et al., 2012). Like other transporter substrates, methylone evokes the release of intraneuronal monoamine neurotransmitters by reversing the normal direction of transporter flux, thereby increasing extracellular concentrations of dopamine, norepinephrine, and 5-HT in the brain (Schindler et al., 2016; Elmore et al., 2017). Heightened levels of serotonin and dopamine potentially contribute to the rewarding properties of methylone (Schindler et al., 2016; Elmore et al., 2017).

Although the popularity of methylone peaked from 2011 to 2015, according to seizure reports (NFLIS, 2016), it remains one of the most commonly used synthetic cathinones, along with mephedrone. In some recent studies, methylone has been detected along with other illicit drugs in different matrixes such as oral fluid (Axelsson et al., 2022), wastewater (Brett et al., 2022) (Australian Criminal Intelligence Commission, 2020), urine (Gomila et al., 2022), hair (Palamar et al., 2016; Palamar et al., 2017; Salomone et al., 2017), and nails (Busardò et al., 2020). These data suggest that the pattern of methylone consumption mainly focuses on weekend and nightlife scenarios.

Methylone is typically administered orally as tablets or pills and less frequently *via* intranasal (insufflation), sublingual, intravenous, and rectal routes. According to users, moderate oral doses of methylone range between 100 and 200 mg, with <100 mg considered a low dose and >200 mg a high dose. After a common dose of methylone, acute effects appear within 15–60 min and last 3–5 h, with maximum effects experienced 60–90 min post-ingestion (World Health Organization, 2014). According to the only published human study evaluating the acute effects of methylone in natural conditions, it has been reported that methylone mainly induces euphoria, stimulation, increased sociability, and altered perception (Poyatos et al., 2021). Overall, methylone exhibits a prototypical psychostimulant and empathogenic MDMA profile.

Like other synthetic cathinones, recreational users often consume small doses after the initial large dose to induce prolonged effects; this pattern of use increases the risk of intoxication or overdose (Liakoni et al., 2017; Gomila et al., 2022) and, in the worst case scenario, death (Boulanger-Gobeil et al., 2012; Cawrse et al., 2012; Pearson et al., 2012; Warrick et al., 2012; Carbone et al., 2013; McIntyre et al., 2013; Barrios et al., 2016; Shimomura et al., 2016; deRoux and Dunn, 2017). Common adverse effects associated with intoxication include anxiety, agitation, palpitations, sweating, tremors, seizures, hyperthermia, and vomiting (Karila et al., 2016; Liakoni et al., 2017).

As previously mentioned, methylone was first designed as a potential treatment for depression and Parkinson’s disease. Recently, this therapeutic approach has resurfaced as a proposal to use methylone to treat post-traumatic stress disorder (PTSD), anxiety, and depression (Warner-Schmidt et al., 2023). In an observational study, methylone exhibited promising results, improving the condition of almost all patients with PTSD (Kelmendi et al., 2022). Methylone could follow the path of MDMA, which has demonstrated its efficacy and safety as a potential treatment in combination with psychotherapy for severe PTSD, anxiety, and related psychiatric disorders (Jerome et al., 2020; Wolfson et al., 2020; Mitchell et al., 2021). To exploit the potential of methylone as a therapeutic tool, it is essential to comprehensively clarify its underlying human pharmacology.

To date, the abuse potential of methylone has mainly been explored in self-administration studies in animal models (Watterson et al., 2012; Creehan et al., 2015; Vandewater et al., 2015; Nguyen et al., 2017; Javadi-Paydar et al., 2018), demonstrating reinforcing properties, less potent than those of other synthetic cathinones, such as mephedrone, MDPV (3,4-methylenedioxypyrovalerone), and α-PVP (α-pyrrolidinopentiophenone) (Schindler et al., 2016; Javadi-Paydar et al., 2018). Nevertheless, data regarding its abuse potential in humans remain scarce, and most available data on the human pharmacology of methylone mainly comprises user reports and the above-mentioned observational study (Poyatos et al., 2021). To date, no controlled studies have explored these effects in humans.

In the present study, we aimed to evaluate the abuse potential and acute pharmacological effects of methylone compared with those of MDMA and placebo after oral administration under controlled conditions.

## 2 Methods

### 2.1 Participants

Eighteen healthy participants (15 males, 3 females) were recruited by word of mouth, but only 17 (14 males, 3 females) completed the study (see 3.1 Participants). Before enrollment, participants underwent a medical examination, including an electrocardiogram (ECG) and blood and urine tests. In addition, the participants received training regarding procedures, questionnaires, and psychomotor tests to be used during the experimental sessions. All subjects reported previous recreational drug use, such as methylone or other synthetic cathinones, amphetamines, and/or MDMA, at least twice in the last year and six times in their lifetime. Subjects were excluded if they had a current or recent (3 months prior to inclusion) organic illness or major surgery or a history of mental disorders, including substance use disorder (except for nicotine), according to the Diagnostic and Statistical Manual of Mental Disorders-5.

### 2.2 Study design

This study was designed as a randomized, double-blind, placebo-controlled, crossover clinical trial to compare the pharmacological effects of methylone with those of MDMA and a placebo. The subjects participated in three experimental sessions, separated by a 1-week wash-out period. In each session, participants received either one of the following three oral medications: 200 mg methylone, 100 mg MDMA, and a placebo. The administered dose of methylone was selected after various dose-finding pilot studies evaluating doses of 50 (n = 3), 100 (n = 6), 150 (n = 5), and 200 mg (n = 7, four subjects received two different doses of methylone and placebo; three subjects received methylone, MDMA and placebo and were included in the definitive study). Methylone and MDMA were evaluated and proved to be well-tolerated (Poyatos et al., 2022a). After the pilot studies, we selected the 200 mg dose of methylone that evoked similar subjective effects to the 100 mg dose of MDMA (e.g., high or liking feelings), which is considered a common dose used in previous pharmacological studies. The subjects received compensation for possible inconveniences owing to their participation. Ethical approval was obtained from the local Human Research Ethics Committee (CEI Hospital Germans Trias i Pujol, code PI-19–082). This study was conducted in compliance with Spanish legislation regarding clinical research and the Declaration of Helsinki. This study has been registered at ClinicalTrials.gov (NCT05488171).

### 2.3 Drugs

Methylone hydrochloride and MDMA hydrochloride were acquired from the LGC Standards (Teddington, United Kingdom). The Pharmacy Department of the Hospital Universitari Germans Trias i Pujol was responsible for manufacturing and dispensing identical, white, and opaque soft gelatin capsules of methylone, MDMA, or placebo (maltodextrin). The methylone capsules contained 50 mg of methylone, and MDMA capsules contained 100 mg of MDMA. During each session, participants received five capsules, combining capsules with active substances and a placebo to reach the methylone or MDMA dose.

### 2.4 Procedures

One day prior to any experimental session, participants were tested for coronavirus disease 2019 (COVID-19) by performing a PCR test. Participants arrived at the Clinical Research Unit (UPIC) at 7:45 a.m. on the day of the session after fasting overnight and remained at the facility for approximately 11 h. Upon arrival, participants provided a urine sample to perform a drug urine test (Drug-Screen Multi 10TD Test [Multi-Line], Nal Von Minden, Germany) to detect the presence of drugs of abuse (amphetamine, barbiturate, benzodiazepine, cocaine, MDMA, methamphetamine, morphine, methadone, tricyclic antidepressants, and tetrahydrocannabinol). Participants were requested to abstain from pre-session use of illicit drugs (1 week), alcohol (48 h), and caffeine or xanthines (24 h) (Papaseit et al., 2016; Poyatos et al., 2021).

To establish levels of methylone, MDMA and their metabolites, blood and oral fluid samples were collected at baseline and 0.25, 0.5, 0.75, 1, 1.5, 2, 3, 4, 6, 8, 10, and 24 h after administration. Urine samples were collected at various time points throughout the session until 24 h (0–4 h, 4–8 h, 8–12 h, 12–24 h). Data on methylone and MDMA concentrations from this study are not presented, although the pharmacokinetics of methylone at doses ranging from 50 to 200 mg from pilot studies have been published (see study design) (Poyatos et al., 2022a).

### 2.5 Physiological effects

Non-invasive systolic and diastolic blood pressure, heart rate, and oral temperature were repeatedly recorded at baseline (−30 and −15 min) and 0.25, 0.50, 0.75, 1, 1.5, 2, 3, 4, 6, 8, 10, and 24 h after administration using a vital signs monitor (Philips Sure Signs VM4 monitors, Phillips, Amsterdam, Netherlands). Pupil diameter was measured using a Haab pupil gauge as a reference under constant light conditions. Electrocardiogram was continuously monitored during the sessions for safety reasons.

### 2.6 Subjective effects

Subjective effects were assessed using visual analog scales (VAS), the short form of the Addiction Research Center Inventory (ARCI), the Evaluation of Subjective Effects of Substances with Abuse Potential questionnaire (VESSPA-SSE), the Sensitivity to Drug Reinforcement Questionnaire (SDRQ), and a pharmacological identification class questionnaire.

VAS allowed participants to rate several adjectives from “not at all” (0 mm) to “extremely” (100 mm) according to their sensations. This instrument contained 31 items, including intensity (any effect), stimulated, high, good effects, bad effects, liking, changes in distances, changes in colors, changes in shapes, changes in lights, hallucinations (seeing lights or spots), hallucinations (seeing things, animals, insects, or people), changes in hearing, hallucinations (hearing sounds or voices), drowsiness, concentration, dizziness, confusion, different or changed body feeling, unreal body feeling, different surroundings, unreal surroundings, open, trust, feeling close to others, I want to be with other people, I want to hug someone, sexual desire, and sexual arousal (Papaseit et al., 2016; Kuypers et al., 2018; Poyatos et al., 2021).

The ARCI 49-item short form is a validated inventory developed to evaluate the subjective effects of various substances, following five subscales: pentobarbital-chlorpromazine-alcohol group (PCAG) measures sedation, morphine-benzedrine group (MBG) measures euphoria, lysergic acid diethylamide (LSD) measures dysphoria and somatic symptoms, benzedrine (BG) measures intellectual efficiency and energy, and amphetamine (A) measures amphetamine-like effects (Lamas et al., 1994; Papaseit et al., 2016; Poyatos et al., 2021).

The standardized VESSPA-SSE questionnaire was used to evaluate the subjective effects of stimulant drugs, such as MDMA. This questionnaire is divided into six subscales that assess sedation (S), psychosomatic anxiety (ANX), changes in perception (CP), pleasure and sociability (SOC), activity and energy (ACT), and psychotic symptoms (PS) (Poudevida et al., 2003; Papaseit et al., 2016; Poyatos et al., 2021).

In addition, participants completed the SDRQ (Kuypers et al., 2018), rating “How pleasant was the substance” (drug liking) and “How much you wanted to use it in that moment” (drug wanting) on a scale of 1–5.

In the pharmacological identification class questionnaire, participants were required to select which pharmacological class better described the administered substance. The options included placebo, benzodiazepines (such as diazepam), alcohol, stimulants (such as amphetamine), designer drugs (such as ecstasy), cocaine, hallucinogens (such as LSD), cannabinoids (such as cannabis), ketamine (special K), GHB (gamma-hydroxybutyric acid; liquid ecstasy), and others (Papaseit et al., 2016).

VAS (except sexual desire and sexual arousal) were performed at baseline and 0.25, 0.50, 0.75, 1, 1.5, 2, 3, 4, 6, 8, 10, and 24 h, but scales regarding intensity (any effect), stimulated, high, good effects, bad effects, and liking were also performed at 2.5 h. SDRQ and VAS regarding sexual desire and arousal were performed at baseline and 1 and 10 h. ARCI and VESSPA-SSE were performed at baseline and 1, 2, 3, 4, 6, 8, and 10 h. The pharmacological class identification questionnaire was performed at 8 h. Subjects were evaluated for psychiatric symptoms using the Young Mania Rating Scale at baseline, 0.5, 1, 4, 6, and 24 h after administration.

### 2.7 Psychomotor performance

Psychomotor performance was evaluated using a specific computerized psychomotor vigilance task (PVT) and a Maddox wing device. The PVT test was performed using software (PC-PVT 2.0) that quantifies the simple reaction time to a numeric stimulus (Reifman et al., 2018). The Maddox wing measures heterophoria due to extraocular muscle imbalance and quantifies exophoria as an indicator of extraocular musculature relaxation and esophoria; this has been previously reported in other studies evaluating the pharmacological effects of psychostimulants (Papaseit et al., 2016). The PVT test was conducted at baseline and 1 and 2 h, while Maddox wing assessments were performed at baseline and 0.25, 0.5, 0.75, 1, 1.5, 2, 3, 4, 6, 8, 10, and 24 h.

### 2.8 Statistical analysis

The sample size was calculated following the methodology of bioequivalence studies considering an alpha risk of 0.05, a power of 80%, and a variability of 30% with a difference of at least 40% between methylone or MDMA and placebo in systolic and diastolic blood pressure. This method resulted in a sample size of at least 10 participants, which was increased to 17 to improve statistical power.

For statistical analysis, values of physiological and subjective effects and psychomotor performance were baseline-adjusted. The maximum effects from baseline (0 h) to 6 h (Emax) and the time needed to achieve maximum effects (Tmax) were determined, and the area under the curve (AUC_0–6h_) was calculated using the trapezoidal rule. One-way repeated measures analysis of variance (ANOVA), with treatment as a factor, assessed differences among the three groups considering Emax and AUC values. If significant differences were detected between treatments, *post hoc* Tukey’s multiple comparison test was performed. For Tmax values, differences were assessed using the non-parametric Friedman test. A Wilcoxon signed-rank test was performed for variables with significant differences in the previous test, with the *p*-value for multiple comparisons adjusted using the Bonferroni test.

The time course (0–10 h) of effects was compared using a two-way repeated measures ANOVA with time and treatment as factors. When significant differences between treatments or in the treatment × time interaction were detected, a *post hoc* Tukey’s multiple comparison test was performed to assess differences between treatments at each time point.

Statistical tests were performed using PASW Statistics, version 18 (SPSS Inc., Chicago, IL, United States ). A *p*-value <0.05 indicated a statistically significant difference.

## 3 Results

### 3.1 Participants

Considering the participants who completed the study, 17 subjects had a mean age of 23 years (range 21–25 years) and a mean weight of 70.8 kg (range 52.1–84.1 kg), presenting a mean body mass index of 23.2 kg/m^2^ (range 17.6–31.3 kg/m^2^). The participants reported previous use of MDMA (100%), poppers (100%), cannabis (94%), mushrooms (71%), amphetamines (41%), cocaine (47%), LSD (18%), ketamine (18%), sedatives/hypnotics (18%), nitrous oxide (12%), opioids (6%), dimethyltryptamine (DMT) (6%), and cathinones (6%). All subjects tested negative on performing urine drug tests, conducted using urine samples collected prior to the study session.

One subject was excluded after completing two experimental sessions owing to undesirable effects, mainly anxiety and uneasiness, attributed to the treatment administered in the second session. Treatment unblinding was performed after excluding the subject, revealing that placebo and MDMA were administered during the first and second treatment sessions, respectively. Side effects that dissipated 1.5 h after appearance were attributed to MDMA, with no therapeutic intervention required to mitigate these effects.

Except for the one excluded subject, all participants completed their experimental sessions without reporting any serious adverse effects, including psychiatric symptoms, psychotic episodes, or hallucinations.

### 3.2 Pharmacological effects

Table 1 summarizes the results (Emax and Tmax) of physiological effects, subjective effects, and psychomotor performance for which the statistical analysis revealed significant differences between the three examined treatments. The results of AUC_0–6h_ and time-course with significant differences between conditions are presented in Supplementary Table S1.

**TABLE 1:** Summary of results (n = 17; mean ± standard deviation; median, range) on physiological measures, subjective effects, and psychomotor performance with statistically significant differences between the three administered conditions (methylone 200 mg, MDMA 100 mg and placebo).

		Methylone	MDMA	Placebo	ANOVA		Tukey/Wilcoxon
					F/X^2^	*p*	
Physiological effects							
**Systolic blood pressure**	Emax (mmHg)	37.35 ± 10.47	31.88 ± 15.28	−8.97 ± 7.64	89.589	<0.001	B,C
	Tmax (h)	0.75 (0.5–1.5)	1 (0.5–1.5)	4 (0.5–6)	17.844	<0.001	B,C
**Diastolic blood pressure**	Emax (mmHg)	12.29 ± 14.31	13.26 ± 13.02	0.47 ± 10.02	5.375	0.010	b,c
	Tmax (h)	1 (0.5–6)	1 (0.75–6)	1 (0.25–6)	0.677	0.713	NS
**Heart rate**	Emax (bpm)	29.38 ± 18.16	21.53 ± 22.17	0.97 ± 12.54	13.778	<0.001	B,C
	Tmax (h)	0.75 (0.5–3)	1 (0.25–6)	3 (0.25–6)	8.848	0.012	B
**Pupil diameter**	Emax (mm)	2.50 ± 0.66	3.10 ± 1.05	−0.15 ± 0.34	95.149	<0.001	B,C
	Tmax (h)	0.75 (0.5–1)	1 (0.5–2)	0 (0–4)	14.323	0.001	A
Visual Analog Scales (VAS)							
**Intensity**	Emax (mm)	45.41 ± 23.56	52.76 ± 30.07	1.12 ± 3.66	30.439	<0.001	B,C
	Tmax (h)	0.75 (0.5–1)	1 (0–2)	0 (0–1)	24.862	<0.001	a,B,C
**Stimulated**	Emax (mm)	48.65 ± 25.47	50.94 ± 32.87	1.29 ± 3.93	25.805	<0.001	B,C
	Tmax (h)	0.75 (0.5–1)	1 (0–2)	0 (0–1)	25.000	<0.001	a,B,C
**High**	Emax (mm)	54.65 ± 29.74	58.71 ± 29.78	1.06 ± 4.12	38.121	<0.001	B,C
	Tmax (h)	0.75 (0.5–1)	1 (0–2)	0 (0–1)	27.692	<0.001	a,B,C
**Good effects**	Emax (mm)	54.35 ± 30.00	57.53 ± 31.01	1.12 ± 4.36	34.635	<0.001	B,C
	Tmax (h)	0.75 (0.5–1)	1 (0–2)	0 (0–1)	26.226	<0.001	a,B,C
**Bad effects**	Emax (mm)	3.82 ± 7.61	11.06 ± 21.72	0	4.204	0.024	c
	Tmax (h)	0 (0–1.5)	0 (0–2)	0 (0–0)	9.920	0.007	NS
**Liking**	Emax (mm)	57.59 ± 31.38	57.18 ± 33.72	1.88 ± 7.51	30.962	<0.001	B,C
	Tmax (h)	0.75 (0.5–1)	1 (0–2.5)	0 (0–1)	25.803	<0.001	a,B,C
**Changes in distances**	Emax (mm)	7.29 ± 13.81	24.35 ± 34.45	0	5.515	0.009	C
	Tmax (h)	0 (0–1)	1 (0–6)	0 (0–0)	12.050	0.002	c
**Changes in lights**	Emax (mm)	11.06 ± 18.28	18.41 ± 25.25	0.53 ± 2.18	4.869	0.014	c
	Tmax (h)	0 (0–1)	0 (0–1.5)	0 (0–1.5)	8.486	0.014	NS
**Focused**	Emax (mm)	19.65 ± 18.05	15.06 ± 17.18	1.24 ± 5.09	8.500	0.001	B,c
	Tmax (h)	0.75 (0–1.5)	1 (0–4)	0 (0–1)	15.709	<0.001	B,c
**Dizziness**	Emax (mm)	10.12 ± 12.36	26.71 ± 26.23	0	11.580	<0.001	a,C
	Tmax (h)	0.5 (0–1.5)	0.75 (0–2)	0 (0–0)	16.128	<0.001	B,C
**Confusion**	Emax (mm)	10.71 ± 21.58	17.29 ± 22.83	0	6.165	0.005	C
	Tmax (h)	0 (0–1)	0.5 (0–2)	0 (0–0)	13.818	0.001	c
**Different body feeling**	Emax (mm)	32.65 ± 29.47	34.35 ± 34.15	1.18 ± 4.60	10.725	<0.001	B,C
	Tmax (h)	0.75 (0–1.5)	1 (0–2)	0 (0–1)	14.913	0.001	B,c
**Different surroundings**	Emax (mm)	10.35 ± 16.93	16.71 ± 26.37	0	4.311	0.022	c
	Tmax (h)	0 (0–1)	0 (0–1.5)	0 (0–0)	10.294	0.006	b,c
**Open**	Emax (mm)	44.82 ± 36.34	38.71 ± 31.45	1.53 ± 6.31	16.175	<0.001	B,C
	Tmax (h)	0.75 (0–1.5)	1.5 (0–2)	0 (0–1)	21.541	<0.001	B,C
**Trust**	Emax (mm)	43.71 ± 35.51	37.59 ± 31.27	1.53 ± 6.31	15.340	<0.001	B,C
	Tmax (h)	0.75 (0–1)	1 (0–3)	0 (0–1)	22.836	<0.001	a,B,C
**Feeling close to others**	Emax (mm)	41.06 ± 35.88	35.53 ± 32.48	1.53 ± 6.31	13.123	<0.001	B,C
	Tmax (h)	0.75 (0–1.5)	1 (0–3)	0 (0–1)	21.849	<0.001	a,B,C
**I want to be with other people**	Emax (mm)	46.41 ± 38.37	46.00 ± 35.10	1.53 ± 6.31	18.333	<0.001	B,C
	Tmax (h)	0.75 (0–1.5)	1 (0–2)	0 (0–1)	21.055	<0.001	B,C
**I want to hug someone**	Emax (mm)	34.71 ± 38.89	38.41 ± 35.05	1.06 ± 4.37	12.162	<0.001	B,C
	Tmax (h)	0.75 (0–1.5)	1 (0–2)	0 (0–1)	15.647	<0.001	B,C
Sensitivity to Drug Reinforcement Questionnaire (SDRQ)							
**How pleasant was the substance**	Emax (score)	2.53 ± 1.37	2.29 ± 1.45	0.24 ± 0.75	22.501	<0.001	B,C
	Tmax (h)	1 (0–1)	1 (0–1)	0 (0–1)	22.933	<0.001	B,C
**How much you wanted to use it in that moment**	Emax (score)	2.29 ± 1.36	1.71 ± 1.57	0.18 ± 0.73	15.221	<0.001	B,C
	Tmax (h)	1 (0–1)	1 (0–1)	0 (0–1)	21.875	<0.001	B,c
Addiction Research Center Inventory (ARCI)							
**ARCI PCAG**	Emax (score)	0.47 ± 4.11	3.35 ± 3.28	0.12 ± 0.99	9.797	<0.001	A,C
	Tmax (h)	1 (1–4)	2 (0–4)	0 (0–1)	21.390	<0.001	B,C
**ARCI MBG**	Emax (score)	7.76 ± 4.93	7.29 ± 5.03	0.47 ± 1.70	21.788	<0.001	B,C
	Tmax (h)	1 (0–1)	1 (0–3)	0 (0–1)	24.875	<0.001	B,C
**ARCI LSD**	Emax (score)	1.88 ± 2.29	2.18 ± 2.16	−0.35 ± 0.61	11.837	<0.001	B,C
	Tmax (h)	1 (0–2)	1 (0–2)	0 (0–1)	19.395	<0.001	B,C
**ARCI BG**	Emax (score)	2.71 ± 2.42	1.06 ± 2.77	0.53 ± 1.28	7.042	0.003	a,B
	Tmax (h)	1 (1–3)	1 (0–4)	0 (0–1)	23.216	<0.001	B,C
**ARCI A**	Emax (score)	4.24 ± 2.25	4.18 ± 2.72	0.47 ± 1.07	22.899	<0.001	B,C
	Tmax (h)	1 (1–1)	1 (0–2)	0 (0–1)	21.814	<0.001	B,C
Subjective Effects of Substances with Abuse Potential (VESSPA-SSE)							
**VESSPA S**	Emax (score)	0.52 ± 0.62	1.03 ± 0.98	0.08 ± 0.17	9.987	<0.001	C
	Tmax (h)	1 (0–6)	1 (0–3)	0 (0–4)	7.538	0.023	NS
**VESSPA ANX**	Emax (score)	1.66 ± 0.96	1.59 ± 0.98	0.13 ± 0.39	28.771	<0.001	B,C
	Tmax (h)	1 (1–3)	1 (0–3)	0 (0–3)	18.429	<0.001	b,C
**VESSPA CP**	Emax (score)	0.07 ± 0.17	0.30 ± 0.45	0	5.785	0.007	a,C
	Tmax (h)	0 (0–1)	0 (0–1)	0 (0–0)	10.667	0.005	c
**VESSPA SOC**	Emax (score)	1.90 ± 1.64	1.81 ± 1.42	0.15 ± 0.53	13.473	<0.001	B,C
	Tmax (h)	1 (0–1)	1 (0–3)	0 (0–1)	22.545	<0.001	B,C
**VESSPA ACT**	Emax (score)	1.82 ± 1.22	1.56 ± 1.35	0.10 ± 0.40	15.926	<0.001	B,C
	Tmax (h)	1 (0–2)	1 (0–3)	0 (0–1)	24.400	<0.001	B,C
**VESSPA PS**	Emax (score)	0.28 ± 0.39	0.33 ± 0.48	0.03 ± 0.12	5.094	0.012	b,c
	Tmax (h)	1 (0–1)	1 (0–2)	0 (0–1)	13.550	0.001	B,c
Psychomotor performance							
**Mean reaction time**	Emax (ms)	−14.63 ± 31.09	36.40 ± 58.70	22.53 ± 39.48	6.818	0.003	A,b
	Tmax (h)	2 (1–2)	1 (1–2)	2 (1–2)	2.533	0.282	NS
**Maddox wing**	Emax (diopter)	−0.97 ± 1.91	−1.69 ± 2.05	0.13 ± 0.43	7.070	0.003	C
	Tmax (h)	0.75 (0–1.5)	1 (0–2)	0 (0–6)	9.088	0.011	a

Abbreviations: ARCI PCAG (sedation), MBG (euphoria), LSD (dysphoria), BG (intellectual efficiency), and A (amphetamine-like effects), VESSPA-SSE S (sedation), ANX (psychosomatic anxiety), CP (changes in perception), SOC (pleasure and sociability), ACT (activity and energy), and PS (psychotic symptoms), ms (milliseconds). Emax is maximum effects from baseline to 6 h, expressed as mean ± standard deviation. Tmax is the time needed to reach maximum effects, expressed as median (range). Differences among Emax were calculated with a one-way ANOVA (degrees of freedom 2 and 32 for all variables) and *post hoc* Tukey’s multiple comparisons test. Differences among Tmax were calculated with Friedman’s test and Wilcoxon signed rank test adjusted for multiple comparisons. Statistical differences in Emax between conditions are indicated as: “a” (*p* < 0.05) or “A” (*p* < 0.01) for methylone vs. MDMA, “b” (*p* < 0.05) or “B” (*p* < 0.01) for methylone vs. placebo, “c” (*p* < 0.05) or “C” (*p* < 0.01) for MDMA vs. placebo. Statistical differences in Tmax between conditions are indicated as: “a” (*p* < 0.016) or “A” (*p* < 0.003) for methylone vs. MDMA, “b” (*p* < 0.016) or “B” (*p* < 0.003) for methylone vs. placebo, “c” (*p* < 0.016) or “C” (*p* < 0.003) for MDMA vs. placebo.

#### 3.2.1 Physiological effects

**FIGURE 1:**
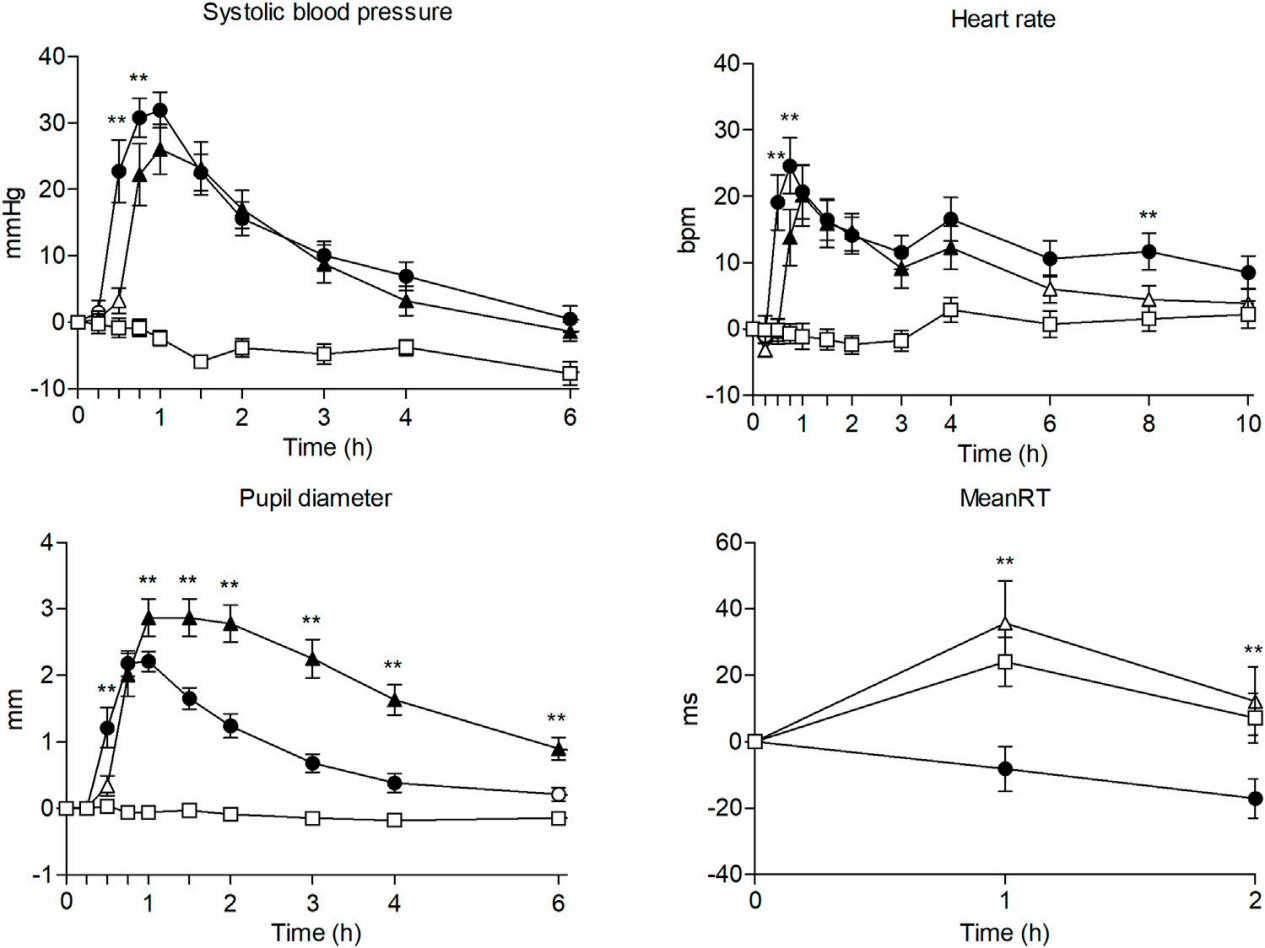
Time course (n = 17; mean ± standard error) of physiological effects and psychomotor performance following oral administration of 200 mg of methylone (○), 100 mg of MDMA (Δ) and placebo (□). Significant differences between methylone and MDMA are represented with *p < 0.05 and **p < 0.01. Significant differences from placebo are represented with filled symbols (*p* < 0.05).

Compared with the placebo, 200 mg methylone and 100 mg MDMA could significantly increase systolic blood pressure, diastolic blood pressure, heart rate, and pupil diameter (see Table 1; Figure 1). The maximum effects of methylone and MDMA on cardiovascular parameters and pupil diameter differed significantly from those of placebo; however, these differences were not observed between active treatments.

Regarding systolic blood pressure and heart rate, methylone administration afforded higher and earlier maximum effects than MDMA administration, although these differences were not statistically significant. However, we noted significant differences between methylone and MDMA considering certain time-course points of systolic blood pressure, heart rate, pupil diameter, AUC, and time to reach maximum effects (Tmax) of pupil diameter, with methylone demonstrating lower AUC and earlier time to reach maximum effects than MDMA.

Methylone produced a sustained increase in heart rate, which differed significantly from placebo until 10 h post-administration, returning to baseline after 24 h. Changes in diastolic blood pressure were markedly similar between methylone and MDMA, considering maximum effects, time to achieve maximum effects, and differences in the time course of effects.

Except at one time-course point (2 h), there were no significant differences in any temperature parameter between active treatments.

#### 3.2.2 Subjective effects

**FIGURE 2:**
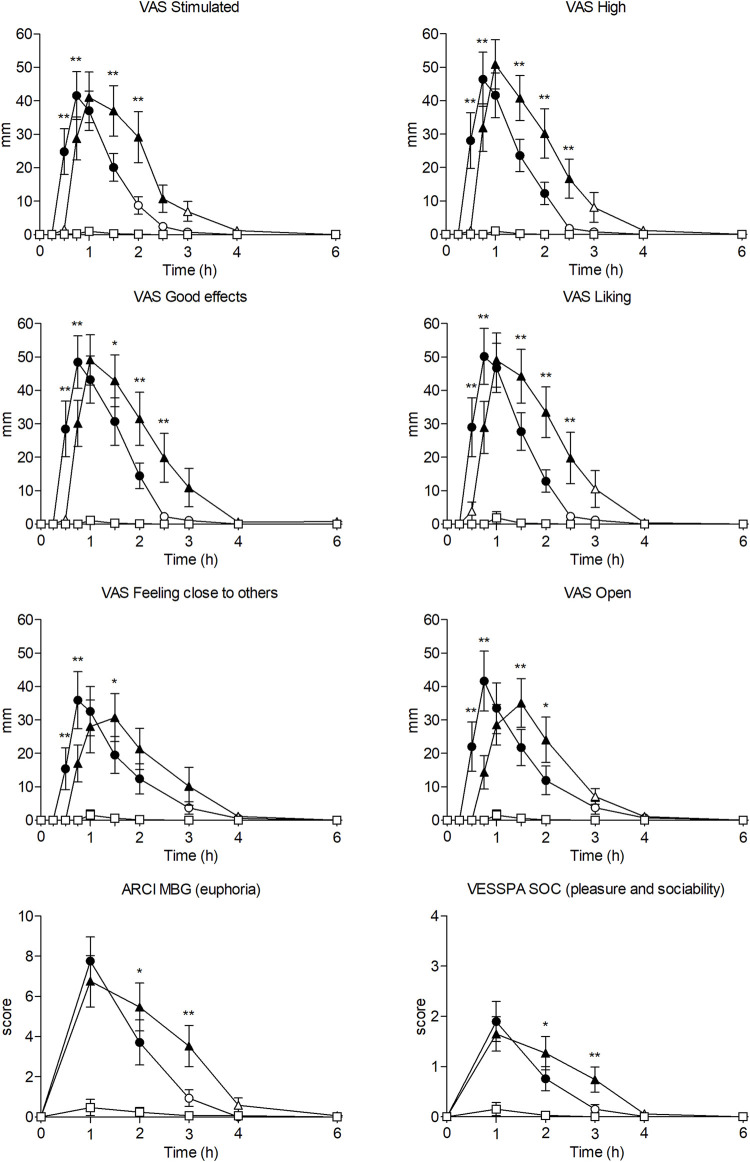
Time course (n = 17; mean ± standard error) of subjective effects following oral administration of 200 mg of methylone (○), 100 mg of MDMA (Δ) and placebo (□). Significant differences between methylone and MDMA are represented with *p < 0.05 and **p < 0.01. Significant differences from placebo are represented with filled symbols (*p* < 0.05).

Compared with the placebo, methylone and MDMA induced significant changes in subjective effects measured using VAS and questionnaires (ARCI, VESSPA, and SDRQ) (Table 1; Figure 2).

The significant changes in VAS were mainly related to stimulation and wellbeing (VAS “stimulation”, “high”, “good effects”, “liking”), altered perception (“changes in distances”, “changes in lights”, “different body feeling”, “different surroundings”), and empathy (“open”, “trust”, “feeling close to others”, “I want to be with other people”, “I want to hug someone”). In general, methylone-induced subjective effects appeared at 0.5 h and normalized at 2.5–3 h post-administration. The subjective effects of MDMA appeared later (0.75 h), returning to baseline at 4 h.

Comparing both active substances, maximum effects and AUCs of most subjective effects measured with VAS did not differ significantly; however, participants who received MDMA reported significantly more ‘dizziness’. Notably, methylone-induced maximum effects appeared significantly earlier (Tmax = 0.75 h), considering effects related to stimulation, wellbeing, and empathy, than those induced by MDMA (Tmax = 1 h). Regarding the time course of effects, we noted significant differences within the first 2.5 h in several scales related to stimulation, wellbeing, altered perception, and empathy between both active treatments, with higher effects of methylone typically documented at earlier time points (0.5–0.75 h), whereas those of MDMA noted at later time points (2–2.5 h) (see Supplementary Table S1).

Methylone and MDMA demonstrated significant changes in all subscales of the ARCI questionnaire, especially MBG (euphoria) and A (amphetamine effects). Methylone generated higher maximum scores on the MBG, BG (intellectual efficiency), and A subscales, whereas MDMA generated higher scores for PCAG and LSD (dysphoria). However, only PCAG (sedation) and BG (intellectual efficiency) revealed significant differences in the maximum effects between both treatments. Comparing the time courses of methylone and MDMA, we noted significant differences at certain time points between 1 and 4 h for ARCI PCAG, MBG, BG, and A.

Compared with placebo, methylone and MDMA demonstrated significant differences in the VESSPA questionnaire in all subscales, particularly in ANX (psychosomatic anxiety), SOC (pleasure and sociability), and ACT (activity and energy). Methylone produced higher maximum effects represented by the VESSPA ANX, SOC, and ACT, whereas MDMA produced higher effects sensitive to the VESSPA S (sedation), CP, and PS subscales. The VESSPA CP was the only subscale with significant differences in the maximum effects and AUC between active treatments. Comparing the time courses of methylone and MDMA, time points between 1 and 3 h differed significantly in VESSPA S, ANX, CP, and SOC.

Regarding SDRQ, methylone was higher rated in the “How pleasant was the substance” and “How much you wanted to use it in that moment” questions than MDMA and placebo, considering maximum effects and AUCs; however, differences among active substances were not statistically significant.

Considering the pharmacological class identification questionnaire, 16 (94.1%) participants identified methylone as a designer drug, similar to MDMA, whereas one (5.9%) participant identified it as a stimulant, similar to amphetamine. After receiving MDMA, 15 (88.2%) participants correctly identified their treatment as a designer drug (MDMA), whereas one (5.9%) subject classified it as a stimulant (amphetamine) and another (5.9%) as a placebo. The placebo was correctly identified by 16 (94.1%) participants, with only one subject classifying it as a stimulant (amphetamine).

#### 3.2.3 Psychomotor performance

In the PVT test, methylone was the only treatment that significantly improved the reaction time by reducing the mean time needed to react to a numeric stimulus (see Table 1). Conversely, MDMA did not impact the reaction time when compared with the placebo. Considering the time course of effects, methylone could significantly decrease the reaction time when compared with MDMA and placebo at 1 and 2 h post-administration.

After methylone and MDMA administration, the participants experienced an inward deviation of the eyes (esophoria) in the Maddox wing device (see Table 1). Notably, MDMA produced higher, but statistically insignificant, levels of esophoria than methylone, considering their maximum effects and AUC. However, methylone administration could induce maximum effects significantly earlier than MDMA administration. During the time course of effects, significant differences were detected between the two active treatments at 2 and 3 h.

## 4 Discussion

To the best of our knowledge, this is the first experimental, controlled study to evaluate the acute pharmacological effects of methylone in humans. Our main finding was that oral doses of 200 mg methylone could induce notable cardiovascular and pleasurable effects, including stimulation, euphoria, wellbeing, increased empathy, and altered perception. Methylone exhibited an effect profile comparable to that of MDMA, with a faster onset and earlier disappearance of subjective effects than MDMA. Typically, the abuse potential of methylone follows the trend of other cathinones, such as natural cathinone and mephedrone (Poyatos et al., 2022b). MDMA exhibited effects similar to those previously reported in other published human studies (Camí et al., 2000; Kirkpatrick et al., 2014; Papaseit et al., 2016; Kuypers et al., 2017; Holze et al., 2020; Studerus et al., 2021).

Our results corroborate those of an observational study in which the effects of methylone were evaluated under natural conditions (Poyatos et al., 2021). In the previous observational study, eight subjects self-administered an oral dose of methylone (100–300 mg, mean dose of 187 mg), which were self-selected according to their preferences and previous experiences. Under the same setting, six subjects were selected and self-administered oral MDMA (75–100 mg, mean dose of 87 mg). In that study, methylone exhibited a prototypical stimulant profile and empathogenic effects frequently attributed to social drugs, such as MDMA. Moreover, methylone was shown to induce a high and sustained increase in the heart rate, also observed under controlled conditions. Regarding subjective effects, methylone induced milder effects than MDMA in the earlier discussed naturalistic study. According to our present results, participants reported higher subjective effects after methylone administration than those documented in the previous study. This discrepancy could be attributed to the wider experience with psychostimulant use among volunteers in the naturalistic study or the distinct settings in which the studies were conducted. In addition, the maximum subjective effects of methylone appeared earlier under controlled conditions.

Furthermore, comparing our results with other synthetic cathinones, such as mephedrone, considered one of the most popular cathinones for recreational use, can be interesting. The human pharmacology of mephedrone was evaluated in a clinical trial including 12 subjects well-experienced in psychostimulant use, who received one oral dose of 200 mg mephedrone compared with 100 mg MDMA and placebo. Methylone and mephedrone appear to share similarities in their physiological effects, given that both could produce higher maximum effects on heart rate and a lower increase in pupil diameter than MDMA. Overall, both cathinones affected vital signs with an early onset and similar intensity. Like methylone, mephedrone exhibits an MDMA-like profile with desirable subjective effects, comprising stimulation, a sensation of wellbeing, altered perception, and increased sociability (Papaseit et al., 2016).

Moreover, the effects of methylone are consistent with the euphorigenic and stimulant effects of *khat*, a naturally occurring cathinone (Brenneisen et al., 1990; Widler et al., 1994).

Interestingly, methylone displayed characteristics that differed from those of MDMA. Unlike MDMA and mephedrone, methylone could induce a sustained and significant increase in heart rate when compared with the placebo, which persisted for 10 h despite the normalization of blood pressure. Moreover, methylone improved psychomotor performance by reducing reaction time. This improvement has also been documented with amphetamine administration (Silber et al., 2006), although there is no clear evidence for MDMA (Camí et al., 2000; Farré et al., 2004; Ramaekers and Kuypers, 2006; Kuypers et al., 2007). Combined with alcohol, mephedrone could reduce the reaction time when compared with that of alcohol alone by mitigating the sedative effects of alcohol. However, mephedrone alone produced markedly similar effects to the placebo on reaction time (De Sousa Fernandes Perna et al., 2016). However, the main difference between methylone and MDMA is the earlier onset and disappearance of subjective effects induced by the former, a particularity that more closely resembles mephedrone (Papaseit et al., 2016).

Considering the data from the pilot studies, methylone displayed fast pharmacokinetics at oral doses ranging from 50 to 200 mg; specifically, 200 mg methylone achieved maximum plasma concentrations (Cmax) of 604 ng/mL at 2 h (Tmax), with an elimination half-life (t_1/2_) of 6.4 h (Poyatos et al., 2022a). These results suggest that the kinetics of methylone are faster than those of MDMA (Tmax of 2–2.5h, t_1/2_ of 7.7–12 h) (Desrosiers et al., 2013; Hysek et al., 2014; Papaseit et al., 2016) but less rapid than those of mephedrone (Tmax of 1.25, t_1/2_ of 2.15) (Papaseit et al., 2016). The maximum pharmacological effects of methylone appeared earlier than the maximum concentrations, but the short elimination half-life could explain the early dissipation of most subjective effects. This fact can also be observed in other studies evaluating human pharmacology of other cathinones such as mephedrone (Papaseit et al., 2016). In that study, mephedrone and MDMA produced earlier maximum effects compared to their Tmax for blood concentration. Effects for mephedrone peaked at 0.75h, while its concentrations peaked at 1.25 h. In case of MDMA, effects peaked at 0.75–1.25 h whereas maximum concentrations were achieved at 2 h. Our results, along with those from previous studies, confirm that pharmacological effects do not need maximum concentrations of the substance in blood to reach peak effects. Moreover, the concentrations of its metabolite 4-hydroxy-3-methoxy-N-methylcathinone (HMMC) were analyzed, revealing a plasma Cmax of 42.1 ng/mL, Tmax of 1.5 h, and t_1/2_ of 6.3 h on administering 200 mg methylone. However, the psychoactive effects might not be attributed to HMMC concentrations despite its affinity for monoamine transporters (Elmore et al., 2017; Luethi et al., 2019), given the poor brain penetration capacity of hydroxylated metabolites (Centazzo et al., 2021). Data regarding the linearity of methylone remain controversial in animals, with evidence of non-linear behavior after subcutaneous administration in rats (Elmore et al., 2017) and linear pharmacokinetics after oral administration (López-Arnau et al., 2013). However, the pharmacokinetics of methylone seem linear in humans (Poyatos et al., 2022a), contrary to MDMA’s, which has been widely described as non-linear (De La Torre et al., 2000). The pharmacokinetic data are consistent with the reduced potency of methylone at monoamine transporters when compared to MDMA. Additionally, methylone may not penetrate the brain as effectively as MDMA, due to the more polar β-keto group. Regarding methylone’s capacity to penetrate into the brain, previous studies confirmed that methylone cross the blood-brain barrier with a brain-to-plasma ratio of 1.42 (López-Arnau et al., 2013), 4.54 (Štefková et al., 2017) or more than 3 (range 3–12) (Centazzo et al., 2021). Studies for MDMA found brain concentrations 5- to 10-fold higher than those in plasma (Mueller et al., 2009). Methylone seems to need higher blood concentrations to produce comparable pharmacological effects with MDMA.

The limitations of the present study should be noted. The statistical analysis lacked the power to detect significant differences between both active drugs owing to the sample size, despite increasing the size with respect to that initially calculated. In addition, although this study included participants of both sexes, the small number of females was insufficient to explore sex differences in the acute effects of methylone. As only one dose of methylone was examined, our findings could not be extrapolated to higher doses or for establishing a dose-response relationship.

Given its short-lived subjective effects, methylone seems suitable for redosing to extend its duration of action. This postulation is reinforced by high scores in the SDRQ questionnaire regarding the desire to repeat the dose, which was performed 1 h post-administration. At that time point, the subjective effects of methylone were already decreasing in most participants, while those of MDMA were at their highest level. This phenomenon is also exhibited by mephedrone, a drug often readministered owing to its brief effects and rapid pharmacokinetics (Papaseit et al., 2016).

## 5 Conclusion

To the best of our knowledge, the present study is the first controlled study to assess the pharmacological effects of methylone in humans. Methylone enhanced cardiovascular parameters and subjective effects, characterized by stimulation, euphoria, increased sociability, and altered perception. This profile was similar to the prototypical effects associated with MDMA, which differed in onset and duration. Overall, these results suggest that the abuse potential of methylone is similar to that of MDMA in humans, although its shorter subjective effects with faster onset may lead to a redosing pattern of use.

## Data Availability

The original contributions presented in the study are included in the article/Supplementary Material, further inquiries can be directed to the corresponding authors.
