# Supplementary material for: Pharmacological effects of methylone and MDMA in humans
Source: Front Pharmacol. 2023 Feb 17;14:1122861. doi: 10.3389/fphar.2023.1122861 (PMC9981643; doi:10.3389/fphar.2023.1122861)
Supplement: Supplementary file 1 [file DataSheet1.docx]

Supplementary Material

Pharmacological effects of methylone and MDMA in humans

Lourdes Poyatos^1,2^, Clara Pérez-Mañá^1,2*^, Olga Hladun^1,2^, Melani Núñez-Montero^1,2^, Georgina de la Rosa^1,2^, Soraya Martín^1^, Ana Maria Barriocanal^1^, Lydia Carabias^3^, Benjamin Kelmendi^4^, Omayema Taoussi^5^, Francesco Paolo Busardò^5^, Francina Fonseca^6,7^, Marta Torrens^7,8^, Simona Pichini^9^, Magí Farré^1,2*†^, Esther Papaseit^1,2†^

*** Correspondence:** Clara Pérez-Mañá: cperezm.mn.ics@gencat.cat; Magí Farré: mfarre.germanstrias@gencat.cat

**Supplementary Table 1**. Summary of results (n=17; mean ± standard deviation) of AUC and time-course on physiological measures, subjective effects, and psychomotor performance with statistically significant differences between the three administered conditions (methylone 200 mg, MDMA 100 mg and placebo).

|  | | Methylone | MDMA | Placebo | ANOVA | | Tukey |
| --- | --- | --- | --- | --- | --- | --- | --- |
|  |  | **Multiple comparison-Tukey** | | | **F** | **p** |  |
| Physiological effects | | | | | | | |
| Systolic blood pressure | AUC_0-6h_ (mmHg·h) | 69.70 ± 33.70 | 52.80 ± 50.29 | -25.44 ± 24.86 | 29.117 | <0.001 | B,C |
|  | T-C | 0.5^A,B^, 0.75^A,B,C^, 1^B,C^, 1.5^B,C^, 2^B,C^, 3^B,C^, 4^B,c^, 6^B,c^ | | | 16.337 | <0.001 |  |
| Diastolic blood pressure | AUC_0-6h_ (mmHg·h) | 13.10 ± 36.27 | 19.07 ± 28.77 | -3.38 ± 23.35 | 2.574 | 0.092 | NS |
|  | T-C | 0.75^B,C^, 1^B,C^, 1.5^B,C^, 2^B,C^ | | | 6.465 | <0.001 |  |
| Heart rate | AUC_0-6h_ (bpm·h) | 84.21 ± 63.74 | 62.72 ± 63.39 | 0.22 ± 33.86 | 12.423 | <0.001 | B,C |
|  | T-C | 0.5^A,B^, 0.75^A,B,C^, 1^B,C^, 1.5^B,C^, 2^B,C^, 3^B,C^, 4^B,C^, 6^B^, 8^A,B^, 10^b^ | | | 10.237 | <0.001 |  |
| Temperature | AUC_0-6h_ (ºC·h) | 1.13 ± 0.92 | 0.74 ± 1.09 | 0.59 ± 1.37 | 1.347 | 0.274 | NS |
|  | T-C | 1.5^b^, 2^a,B^, 3^B^,4^b^ | | | 1.700 | 0.027 |  |
| Pupil diameter | AUC_0-6h_ (mm·h) | 4.88 ± 2.71 | 10.78 ± 4.74 | -0.67 ± 1.20 | 53.766 | <0.001 | A,B,C |
|  | T-C | 0.5^A,B^, 0.75^B,C^, 1^A,B,C^, 1.5^A,B,C^, 2^A,B,C^, 3^A,B,C^, 4^A,B,C^, 6^A,C^, 8^a,C^ | | | 28.883 | <0.001 |  |
| Visual Analog Scales (VAS) | | | | | | | |
| Intensity | AUC_0-6h_ (mm·h) | 45.09 ± 29.76 | 69.01 ± 53.98 | 0.68 ± 2.45 | 16.204 | <0.001 | B,C |
|  | T-C | 0.5^A,B^, 0.75^a,B,C^, 1^B,C^, 1.5^A,B,C^, 2^A,C^, 2.5^A,C^ | | | 13.507 | <0.001 |  |
| Stimulated | AUC_0-6h_ (mm·h) | 46.75 ± 33.31 | 68.51 ± 57.73 | 0.71 ± 2.31 | 13.284 | <0.001 | B,C |
|  | T-C | 0.5^A,B^, 0.75^A,B,C^, 1^B,C^, 1.5^A,B,C^, 2^A,C^, 2.5^c^ | | | 11.995 | <0.001 |  |
| High | AUC_0-6h_ (mm·h) | 53.69 ± 38.95 | 79.14 ± 61.58 | 0.51 ± 2.03 | 16.768 | <0.001 | B,C |
|  | T-C | 0.5^A,B^, 0.75^A,B,C^, 1^B,C^, 1.5^A,B,C^, 2^A,b,C^, 2.5^A,C^ | | | 13.970 | <0.001 |  |
| Good effects | AUC_0-6h_ (mm·h) | 60.04 ± 44.29 | 83.42 ± 70.58 | 0.63 ± 2.48 | 16.763 | <0.001 | B,C |
|  | T-C | 0.5^A,B^, 0.75^A,B,C^, 1^B,C^, 1.5^a,B,C^, 2^A,B,C^, 2.5^A,C^, 3^c^ | | | 13.078 | <0.001 |  |
| Bad effects | AUC_0-6h_ (mm·h) | 2.18 ± 4.62 | 11.38 ± 24.81 | 0 | 3.441 | 0.044 | c |
|  | T-C | 1^c^, 2^A,C^ | | | 1.612 | 0.036 |  |
| Liking | AUC_0-6h_ (mm·h) | 59.73 ± 42.59 | 83.91 ± 69.81 | 0.94 ± 3.79 | 16.198 | <0.001 | B,C |
|  | T-C | 0.5^A,B^, 0.75^A,B,C^, 1^B,C^, 1.5^A,B,C^, 2^A,b,C^, 2.5^A,C^ | | | 12.748 | <0.001 |  |
| Change in distances | AUC_0-6h_ (mm·h) | 3.80 ± 7.82 | 24.16 ± 39.58 | 0 | 5.207 | 0.011 | a,c |
|  | T-C | 0.75^a,C^, 1^A,C^, 1.5^A,C^ | | | 3.913 | <0.001 |  |
| Change in colors | AUC_0-6h_ (mm·h) | 4.24 ± 9.79 | 11.42 ± 31.99 | 0 | 1.789 | 0.183 | NS |
|  | T-C | 0.75^A,B^, 1^a,C^, 1.5^C^ | | | 2.097 | 0.003 |  |
| Change in shapes | AUC_0-6h_ (mm·h) | 1.21 ± 3.45 | 10.46 ± 28.70 | 0 | 1.967 | 0.156 | NS |
|  | T-C | 1^C^, 1.5^A,C^, 2^a,c^ | | | 2.147 | 0.002 |  |
| Changes in lights | AUC_0-6h_ (mm·h) | 7.18 ± 15.39 | 19.38 ± 30.52 | 0.26 ± 1.09 | 4.443 | 0.020 | c |
|  | T-C | 0.75^a,B,c^, 1^A,B,C^, 1.5^A,C^, 2^c^ | | | 4.404 | <0.001 |  |
| Drowsiness | AUC_0-6h_ (mm·h) | 29.56 ± 55.45 | 39.23 ± 65.39 | 6.43 ± 16.80 | 3.067 | 0.060 | NS |
|  | T-C | 1.5^c^, 2^a,C^, 3^B,C^ | | | 2.020 | 0.005 |  |
| Focused | AUC_0-6h_ (mm·h) | 14.25 ± 18.39 | 16.65 ± 25.09 | 0.64 ± 2.64 | 4.505 | 0.019 | c |
|  | T-C | 0.75^A,B^, 1^B,C^, 1.5^b,C^, 2^A,C^ | | | 4.230 | <0.001 |  |
| Dizziness | AUC_0-6h_ (mm·h) | 8.65 ± 16.87 | 23.21 ± 21.85 | 0 | 10.019 | <0.001 | a,C |
|  | T-C | 0.5^b^, 0.75^b,C^, 1^A,C^, 1.5^A,C^, 2^C^ | | | 5.489 | <0.001 |  |
| Confusion | AUC_0-6h_ (mm·h) | 11.12 ± 27.81 | 19.99 ± 28.09 | 0 | 4.126 | 0.025 | c |
|  | T-C | 0.75^B,C^, 1^B,C^, 1.5^C^, 2^A,C^ | | | 2.714 | <0.001 |  |
| Different body feeling | AUC_0-6h_ (mm·h) | 26.49 ± 29.94 | 42.92 ± 58.24 | 0.46 ± 1.73 | 6.274 | 0.005 | C |
|  | T-C | 0.5^a,B^, 0.75^B,C^, 1^B,C^, 1.5^A,b,C^, 2^c^ | | | 5.258 | <0.001 |  |
| Different surroundings | AUC_0-6h_ (mm·h) | 7.23 ± 15.48 | 19.48 ± 40.01 | 0 | 2.848 | 0.073 | NS |
|  | T-C | 0.75^B^, 1^b,C^, 1.5^A,C^, 2^a,c^ | | | 2.752 | <0.001 |  |
| Open | AUC_0-6h_ (mm·h) | 53.15 ± 52.11 | 58.68 ± 53.04 | 1.00 ± 4.12 | 10.541 | <0.001 | B,C |
|  | T-C | 0.5^A,B^, 0.75^A,B,C^, 1^B,C^, 1.5^A,B,C^, 2^a,b,C^ | | | 10.064 | <0.001 |  |
| Trust | AUC_0-6h_ (mm·h) | 49.95 ± 48.39 | 54.00 ± 57.62 | 0.99 ± 4.06 | 8.371 | 0.001 | B,C |
|  | T-C | 0.5^A,B^, 0.75^A,B,C^, 1^B,C^, 1.5^B,C^, 2^b,C^ | | | 6.653 | <0.001 |  |
| Feeling close to others | AUC_0-6h_ (mm·h) | 48.72 ± 50.52 | 58.08 ± 66.11 | 1.03 ± 4.24 | 7.655 | 0.002 | b,C |
|  | T-C | 0.5^A,B^, 0.75^A,B,C^, 1^B,C^, 1.5^a,B,C^, 2^b,C^, 3^c^ | | | 7.248 | <0.001 |  |
| I want to be with other people | AUC_0-6h_ (mm·h) | 63.41 ± 65.55 | 84.29 ± 99.55 | 1.03 ± 4.24 | 8.090 | 0.001 | b,C |
|  | T-C | 0.5^A,B^, 0.75^A,B,C^, 1^B,C^, 1.5^B,C^, 2^A,B,C^, 3^c^ | | | 9.029 | <0.001 |  |
| I want to hug someone | AUC_0-6h_ (mm·h) | 52.13 ± 70.86 | 61.43 ± 74.37 | 0.69 ± 2.85 | 6.981 | 0.003 | b,C |
|  | T-C | 0.5^a,b^, 0.75^a,B,C^, 1^B,C^, 1.5^B,C^, 2^B,C^, 3^c^ | | | 5.605 | <0.001 |  |
| SDRQ questionnaire | | | | | | |  |
| How pleasant was the substance | AUC_0-10h_ (score·h) | 12.65 ± 6.87 | 11.47 ± 7.24 | 1.18 ± 3.76 | 22.501 | <0.001 | B,C |
|  | T-C | 1^B,C^ | | | 22.501 | <0.001 |  |
| How much you wanted to use it in that moment | AUC_0-10h_ (score·h) | 11.74 ± 7.02 | 8.53 ± 7.86 | 0.88 ± 3.64 | 14.970 | <0.001 | B,C |
|  | T-C | 1^a,B,C^ | | | 15.064 | <0.001 |  |
| ARCI inventory | | | | | | | |
| ARCI PCAG | AUC_0-6h_ (score·h) | 3.21 ± 9.49 | 7.03 ± 9.50 | 0.41 ± 2.69 | 4.684 | 0.016 | c |
|  | T-C | 1^A^, 2^A,C^, 3^C^, 4^b,c^ | | | 2.844 | 0.001 |  |
| ARCI MBG | AUC_0-6h_ (score·h) | 12.41 ± 9.91 | 16.71 ± 13.95 | 0.85 ± 3.27 | 13.912 | <0.001 | B,C |
|  | T-C | 1^B,C^, 2^a,B,C^, 3^A,C^ | | | 12.525 | <0.001 |  |
| ARCI LSD | AUC_0-6h_ (score·h) | 1.50 ± 3.03 | 2.38 ± 4.24 | -0.68 ± 1.31 | 6.309 | 0.005 | C |
|  | T-C | 1^B,C^ | | | 7.280 | <0.001 |  |
| ARCI BG | AUC_0-6h_ (score·h) | 3.47 ± 5.56 | 1.76 ± 6.79 | 1.12 ± 3.01 | 1.260 | 0.297 | NS |
|  | T-C | 1^A,B^, 2^A,B^ | | | 5.377 | <0.001 |  |
| ARCI A | AUC_0-6h_ (score·h) | 6.59 ± 4.80 | 9.97 ± 7.75 | 0.85 ± 1.92 | 13.575 | <0.001 | B,C |
|  | T-C | 1^B,C^, 2^a,B,C^, 3^A,C^ | | | 12.022 | <0.001 |  |
| VESSPA questionnaire | | | | | | | |
| VESSPA S | AUC_0-6h_ (score·h) | 1.31 ± 1.95 | 2.33 ± 2.81 | 0.11 ± 0.26 | 5.671 | 0.008 | C |
|  | T-C | 1^A,C^, 2^a,b,C^, 3^b,C^ | | | 4.236 | <0.001 |  |
| VESSPA ANX | AUC_0-6h_ (score·h) | 4.33 ± 3.56 | 4.35 ± 2.87 | 0.39 ± 1.10 | 14.733 | <0.001 | B,C |
|  | T-C | 1^A,B,C^, 2^B,C^, 3^B,C^, 4^b,C^ | | | 12.397 | <0.001 |  |
| VESSPA CP | AUC_0-6h_ (score·h) | 0.07 ± 0.17 | 0.41 ± 0.66 | 0 | 5.467 | 0.009 | a,c |
|  | T-C | 1^A,C^ | | | 5.442 | <0.001 |  |
| VESSPA SOC | AUC_0-6h_ (score·h) | 2.83 ± 2.54 | 3.75 ± 3.45 | 0.18 ± 0.65 | 11.333 | <0.001 | B,C |
|  | T-C | 1^B,C^, 2^a,B,C^, 3^A,C^ | | | 9.584 | <0.001 |  |
| VESSPA ACT | AUC_0-6h_ (score·h) | 2.72 ± 2.40 | 3.12 ± 2.98 | 0.20 ± 0.81 | 9.083 | 0.001 | B,C |
|  | T-C | 1^B,C^, 2^B,C^, 3^C^ | | | 10.524 | <0.001 |  |
| VESSPA PS | AUC_0-6h_ (score·h) | 0.43 ± 0.72 | 0.73 ± 1.39 | 0.04 ± 0.16 | 3.580 | 0.040 | c |
|  | T-C | 1^B,C^, 2^C^, 3^c^ | | | 3.014 | <0.001 |  |
| Psychomotor performance | | | | | | | |
| Mean reaction time | AUC_0-2h_ (ms·h) | -16.73 ± 38.83 | 41.79 ± 68.89 | 27.59 ± 38.85 | 8.040 | 0.001 | A,b |
|  | T-C | 1^A,B^, 2^A,B^ | | | 4.269 | 0.004 |  |
| Maddox wing | AUC_0-6h_ (diopter·h) | -2.17 ± 5.92 | -4.60 ± 5.21 | 0.26 ± 1.23 | 6.022 | 0.006 | C |
|  | T-C | 0.75^B,c^, 1^B,C^, 1.5^B,C^, 2^A,B,C^, 3^A,C^, 4^c^ | | | 4.606 | <0.001 |  |

Abbreviations: T-C (time course), ARCI PCAG (sedation), MBG (euphoria), LSD (dysphoria), BG (intellectual efficiency), and A (amphetamine-like effects), VESSPA-SSE S (sedation), ANX (psychosomatic anxiety), CP (changes in perception), SOC (pleasure and sociability), ACT (activity and energy), and PS (psychotic symptoms), ms (milliseconds). AUC is area under the curve, expressed as mean ± standard deviation. Differences among AUC were calculated with a one-way ANOVA (degrees of freedom 2 and 32 for all variables) and post-hoc Tukey’s multiple comparisons test. Differences among time course (0-10 h) were calculated with two-way ANOVA and post-hoc Tukey’s multiple comparisons test. The degrees of freedom were 2 and 352 for physiological effects, 2 and 384 for VAS “intensity (any effect)”, “stimulated”, “high”, “good effects”, “bad effects”, and “liking”, 2 and 352 for the rest of VAS, 2 and 64 for SDRQ and psychomotor performance, and 2 and 224 for ARCI and VESSPA. Statistical differences in AUC and time course between conditions are indicated as: “a” (p<0.05) or “A” (p<0.01) for methylone vs MDMA, “b” (p<0.05) or “B” (p<0.01) for methylone vs placebo, “c” (p<0.05) or “C” (p<0.01) for MDMA vs placebo.
